# Transcription Factor E2F7 Hampers the Killing Effect of NK Cells against Colorectal Cancer Cells via Activating RAD18 Transcription

**DOI:** 10.4014/jmb.2308.08026

**Published:** 2023-11-17

**Authors:** Bingdong Jiang, Binghua Yan, Hengjin Yang, He Geng, Peng Li

**Affiliations:** 1 Department of Oncology, Union Jiangbei Hospital Huazhong University of Science and Technology, Wuhan 430100, P.R. China; 2 Department of Radiation Oncology, Huai'an Hospital of Huai'an City, Huai'an City, 223001, P.R. China

**Keywords:** E2F7, RAD18, NK cell killing, colorectal cancer

## Abstract

As a pivotal defensive line against multitudinous malignant tumors, natural killer (NK) cells exist in the tumor microenvironment (TME). RAD18 E3 Ubiquitin Protein Ligase (RAD18) has been reported to foster the malignant progression of multiple cancers, but its effect on NK function has not been mined. Here, the study was designed to mine the mechanism by which RAD18 regulates the killing effect of NK cells on colorectal cancer (CRC) cells. Expression of E2F Transcription Factor 7 (E2F7) and RAD18 in CRC tissues, their correlation, binding sites, and RAD18 enrichment pathway were analyzed by bioinformatics. Expression of E2F7 and RAD18 in cells was assayed by qRT-PCR and western blot. Dual-luciferase assay and chromatin immunoprecipitation (ChIP) assay verified the regulatory relationship between E2F7 and RAD18. CCK-8 assay was utilized to assay cell viability, colony formation assay to detect cell proliferation, lactate dehydrogenase (LDH) test to assay NK cell cytotoxicity, ELISA to assay levels of granulocyte-macrophage colony-stimulating factor (GM-CSF), tumor necrosis factor-α (TNF-α) and interferon-γ (IFN-γ), and immunofluorescence to detect expression of toxic molecules perforin and granzyme B. High expression of RAD18 and E2F7 was found in CRC tissues and cells. Silencing RAD18 could hamper the proliferation of CRC cells, foster viability and cytotoxicity of NK cells, and increase the secretion of GM-CSF, TNF-α, IFN-γ as well as the expression of perforin and granzyme B. Additionally, ChIP and dual-luciferase reporter assay ascertained the binding relationship between RAD18 promoter region and E2F7. E2F7 could activate the transcription of RAD18, and silencing RAD18 reversed the inhibitory effect of E2F7 overexpression on NK cell killing. This work clarified the inhibitory effect of the E2F7/RAD18 axis on NK cell killing in CRC, and proffered a new direction for immunotherapy of CRC in targeted immune microenvironment.

## Introduction

Colorectal cancer (CRC) is the third most common cancer among malignant tumors of the digestive system [1, 2]. Surgery, chemotherapy, radiotherapy, and combination therapy are the standard treatments for CRC [3]. Although CRC treatment has witnessed great progress in recent years, metastatic disease remains challenging with a 5-year survival rate of only 20% [4]. Therefore, developing fresh cure strategies to improve patient survival is in urgent need.

Immunotherapy is a new type of therapy in recent years that fights cancer cells by patient’s own immune system. Featured with specificity and low toxicity, tumor immunotherapy has brought hope for the cure of tumors [3], and functions greatly in clearing tumor cells and infected cells [5]. Among them, natural killer (NK) cells are important in the body’s natural immune system, which can eliminate tumor cells through contacting dependent cytotoxicity and cytokine production for immune regulation [5]. For example, in breast cancer (BC), tumor necrosis factor–α (TNF-α)–induced protein-8 like-2 (TIPE2) fosters the production of interferon-γ (IFN-γ) and TNF-α by NK cells, enhances the killing ability of NK cells to BC cells, and hampers the development and metastasis of BC [6]. Additionally, the vital modulatory role of NK cells in CRC progression has also been reported. For instance, lncRNA small nucleolar RNA host gene 10 (SNHG10) hampers the killing effect of NK cells on CRC cells by up-regulating inhibin subunit beta C (INHBC) expression [7]. Tang *et al*. [8] reported that miR-20a significantly reduced the killing effect of NK cells on CRC cells by inhibiting major histocompatibility complex class I chain-related A (MICA) expression in CRC cells. Therefore, revealing the mechanism of NK cells in CRC was a fresh aspect to find new therapies for curing CRC, which is crucial for CRC immunotherapy targeting the immune microenvironment.

RAD18 E3 Ubiquitin Protein Ligase (RAD18) is a DNA damaging-activated E3 ubiquitin ligase that can act as a key modulator to coordinate homologous recombination repair by monitoring DNA damage signals [9]. RAD18 has been identified to be an oncogene in malignant tumors and is linked with cancer metastasis [10]. For example, RAD18 is up-regulated in human esophageal squamous cell carcinoma (ESCC) and promotes invasion and migration of ESCC cells via the JNK-MMPs pathway [11]. In ovarian cancer (OC), LINC00858 binds miR-134-5p to promote RAD18 expression, thus fostering OC cell migration, proliferation, epithelial-mesenchymal transition and hampering apoptosis [12]. It is worth noting that the effect of RAD18 on NK cell killing has not been reported, and the mechanism of RAD18 in NK cell killing in CRC is still indistinct. Therefore, we intended to mine how RAD18 works to regulate NK cell function in CRC.

In CRC, to clarify the function and modulatory mechanism of RAD18, bioinformatics software was employed, and it was found that RAD18 may regulate NK cell killing in CRC. Therefore, we further analyzed and verified the molecular mechanism by which RAD18 affected the killing effect of NK cells on CRC cells through cellular molecular experiments. These findings may help to exploit new therapeutic strategies for CRC.

## Materials and Methods

### Bioinformatic Analysis

From The Cancer Genome Atlas (TCGA), mRNA expression data of CRC were acquired and the target gene was determined by differential analysis combined with literature citation [13]. After that, GSEA assayed the enrichment pathways of RAD18. ChIPBase and hTFtarget were adopted to predict the upstream transcription factors of RAD18, while the motif sites of RAD18 and upstream transcription factors were predicted by Pearson correlation analysis and JASPAR.

### Cell Culture and Transfection

Human normal colonic epithelial cell line CCD-18Co, human colorectal adenocarcinoma cell lines (LS174T, HCT116, HCT-15, SW480, LOVO), and human renal epithelial cell line 293T were purchased from American Type Culture Collection (ATCC, USA). The above cells were kept in DMEM/F12 medium (Gibco, USA) containing 10% fetal bovine serum (FBS).

Human NK cell line NK92 was bought from ATCC and cultured in Minimum Essential Medium α (MEMα) containing 10% horse serum, 10% FBS, 0.1 mM β-mercaptoethanol, and 1% penicillin-streptomycin in an incubator (37°C, 5% CO_2_) [14]. To activate NK92 cells, they were stimulated with 100 U/ml IL-2 (BD Biosciences, USA) for 24 h.

The oe-E2F7 vector, si-RAD18#1 vector, si-RAD18#2 vector, si-E2F7#1 vector, and si-E2F7#1 vector were purchased from RiboBio (China). Plasmids were transfected into the corresponding CRC cells utilizing the Lipofectamine 2000 kit (Thermo Fisher Scientific, USA).

### NK92 Cells Were Co-Cultured with CRC Cells

CRC cells were diluted to 1.5 × 10^5^ cells/ml in DMEM/F12 containing 10% FBS. NK92 cells activated by IL-2 were co-cultured with CRC cells after different transfection treatments at a ratio of 10:1 effector/target (E: T) for 4h at 37°C. Then, culture supernatant was collected for subsequent experiments [15].

### Cell Viability Assay

Cell viability was assayed by CCK-8 (Dojindo, Japan). Cells were put in 96-well plates, with 10 μl of CCK-8 solution added at 0 h, 24 h, 48 h, and 72 h to each well. Cell absorbance was captured and recorded at 450 nm utilizing a Varioskan Flash system (Thermo Fisher Scientific).

### Cell Proliferation Assay

Cultured in 12-well plates for 10 days, cells were fixed with 4% paraformaldehyde for 15 min and then stained with 0.1% crystal violet for 30 min. Finally, cell proliferation was recorded by camera photography.

### Detection of Cytotoxicity

The cytotoxicity of different treatments was determined by rating the integrity of the plasma membrane utilizing a lactate dehydrogenase (LDH) kit (Beyotime, China). In brief, IL-2-activated NK92 cells were incubated with different transfected cells for 24 h in 96-well plates. After centrifugation, 120 μl of supernatant was gathered and transferred into a new 96-well plate. LDH release reagent was utilized to lyse cells, absorbance was measured at 490 nm utilizing a Varioskan Flash system (Thermo Fisher Scientific) [7].

### ELISA

The supernatants of IL-2-activated NK92 cells co-cultured with CRC cells with different transfection treatments were collected. Levels of IFN-γ, TNF-α and granulocyte-macrophage colony-stimulating factor (GM-CSF) were assayed by human IFN-γ, TNF-α and GM-CSF ELISA kits (Abcam, UK). Absorbance values at 450 nm were determined using a Varioskan Flash system (Thermo Fisher Scientific).

### Immunofluorescence (IF)

Here, IF assays were done in line with the method described by Huang *et al*. [7]. The antibodies, anti-perforin and anti-granzyme B, were from Abcam. Mean fluorescence intensity was determined using ImageJ software.

### qRT-PCR

Total RNA was obtained by the Trizol method, while RNA concentration was measured by spectrophotometer. PrimeScript RT Reagent Kit (Takara, Japan) was used for cDNA synthesis, and subsequently, qRT-PCR was performed using TB Green *Premix* Ex Taq II (Tli RNaseH Plus) (Takara) according to the manufacturer’s instructions. The reference gene was GAPDH. Findings were analyzed and calculated by the 2^−ΔΔCT^ method. The primers are listed in Table 1.

### Western Blot

Western blot was performed according to the method of Sato *et al*. [16]. Total proteins were extracted from cells using RIPA lysate buffer. Equal quantities of proteins (10 μg) were resolved by sodium dodecyl sulfate-polyacrylamide gel electrophoresis and subsequently transferred onto a polyvinylidene fluoride membrane, which was sealed with 5% skim milk in TBST for 1 h and incubated overnight at 4°C utilizing primary antibodies: rabbit anti-RAD18 (1:10000, Abcam, UK), rabbit anti-E2F7 (1:1000, Thermo Fisher). The membranes were then kept with secondary antibody goat anti-rabbit IgG H&L (HRP) (1:2000, Abcam) for 1 h. Immunoreactivity was detected using an ECL system (Thermo Fisher Scientific). The intensities of protein bands were normalized to rabbit anti-β-actin (1:1000, Abcam) and presented as the ratio to the control.

### Dual-Luciferase Gene Reporter Assay

We built pGL3-Basic-RAD18-WT and pGL3-Basic-RAD18-MUT luciferase reporter vectors (Promega, USA). The si-NC and si-E2F7 were co-transfected into 293T cells with the above two plasmids for 48 h, and the luciferase activity of each transfection group was detected by luciferase activity assay kit (Promega).

### Chromatin Immunoprecipitation (ChIP)

ChIP assay was performed according to the previous method [17]. Antibodies used were: anti-E2F7 antibody (Abcam) or anti-igG antibody (Abcam). The primer sets for PCR are listed in Table 2.

### Statistics Analysis

All assays were done three times. Data were expressed as mean ± SD, and statistical analysis was done by GraphPad Prism 6. The differences between groups were compared by t-test or one-way analysis of variance, and the Pearson correlation coefficient was utilized to perform correlation analysis. * means *p* <0 .05, indicating statistical significance.

## Results

### RAD18 Fosters the Proliferation of CRC Cells

**Fig. 1:**
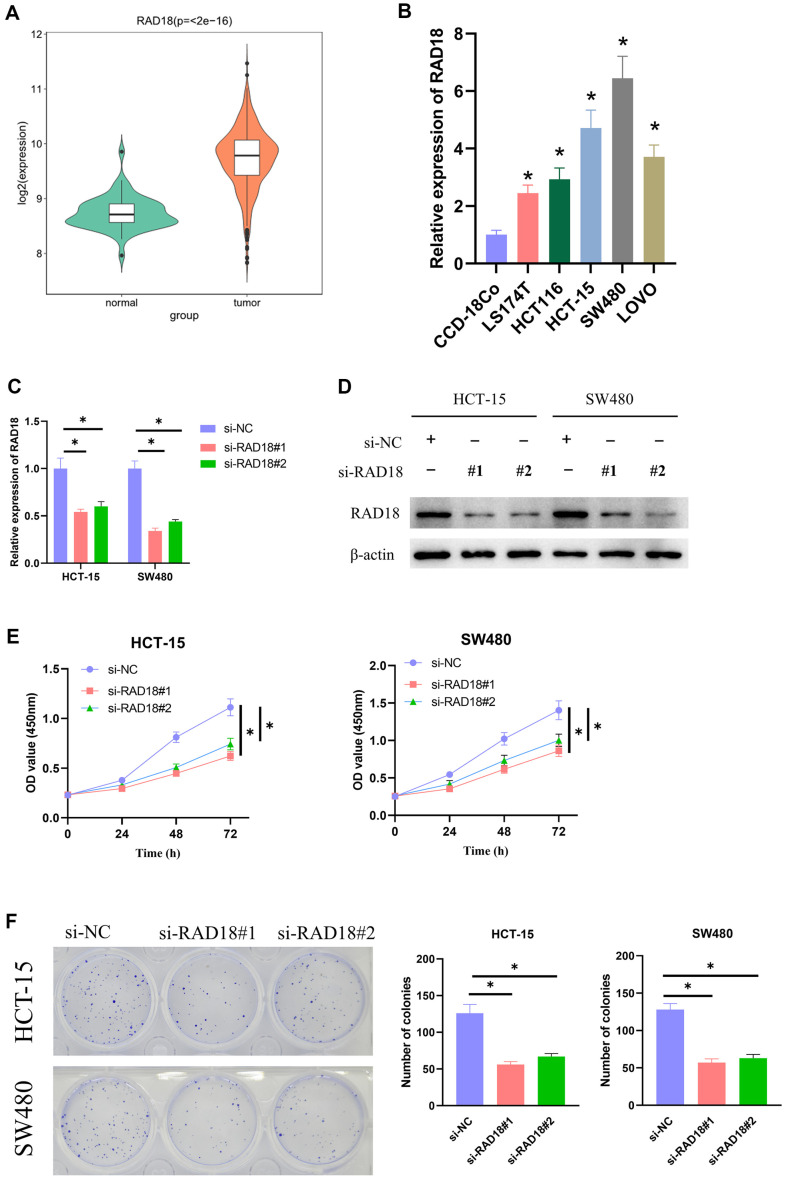
RAD18 fosters the proliferation of CRC cells. (**A**) RAD18 expression underwent bioinformatics analysis; (**B**) Detection of RAD18 expression by qRT-PCR; (**C**) Detection of RAD18 transfection efficiency by qRT-PCR; (**D**) Detection of protein expression of RAD18 by western blot; (**E**) The viability of transfected cells was detected by CCK-8 assay; (**F**) The proliferation ability of transfected cells was detected by colony formation assay. * means *p* < 0.05.

RAD18 up-regulation was revealed by bioinformatics analysis in CRC tissues (Fig. 1A). Significant upregulation of RAD18 in CRC cell lines was shown by qRT-PCR results in contrast with human normal colon mucosal epithelial cells (Fig. 1B). According to RAD18 expression in CRC cells, HCT-15 and SW480 cells were selected to construct the cell model of RAD18 knockdown expression. As the results of qRT-PCR and western blot revealed, si-RAD18#1 and si-RAD18#2 significantly reduced the expression of RAD18 in HCT-15 and SW480 cells (Fig. 1C and 1D). Cell viability was then measured using CCK-8, and si-RAD18#1 and si-RAD18#2 greatly reduced HCT-15 and SW480 cell viability (Fig. 1E). Cell proliferation was measured by colony formation assay, and si-RAD18#1 and si-RAD18#2 significantly reduced the proliferation capacity of HCT-15 and SW480 cells (Fig. 1F). In conclusion, RAD18 had significantly high expression in CRC tissues and cells and could foster CRC cells to proliferate.

### RAD18 Hampers the Killing Effect of NK Cells on CRC Cells

**Fig. 2:**
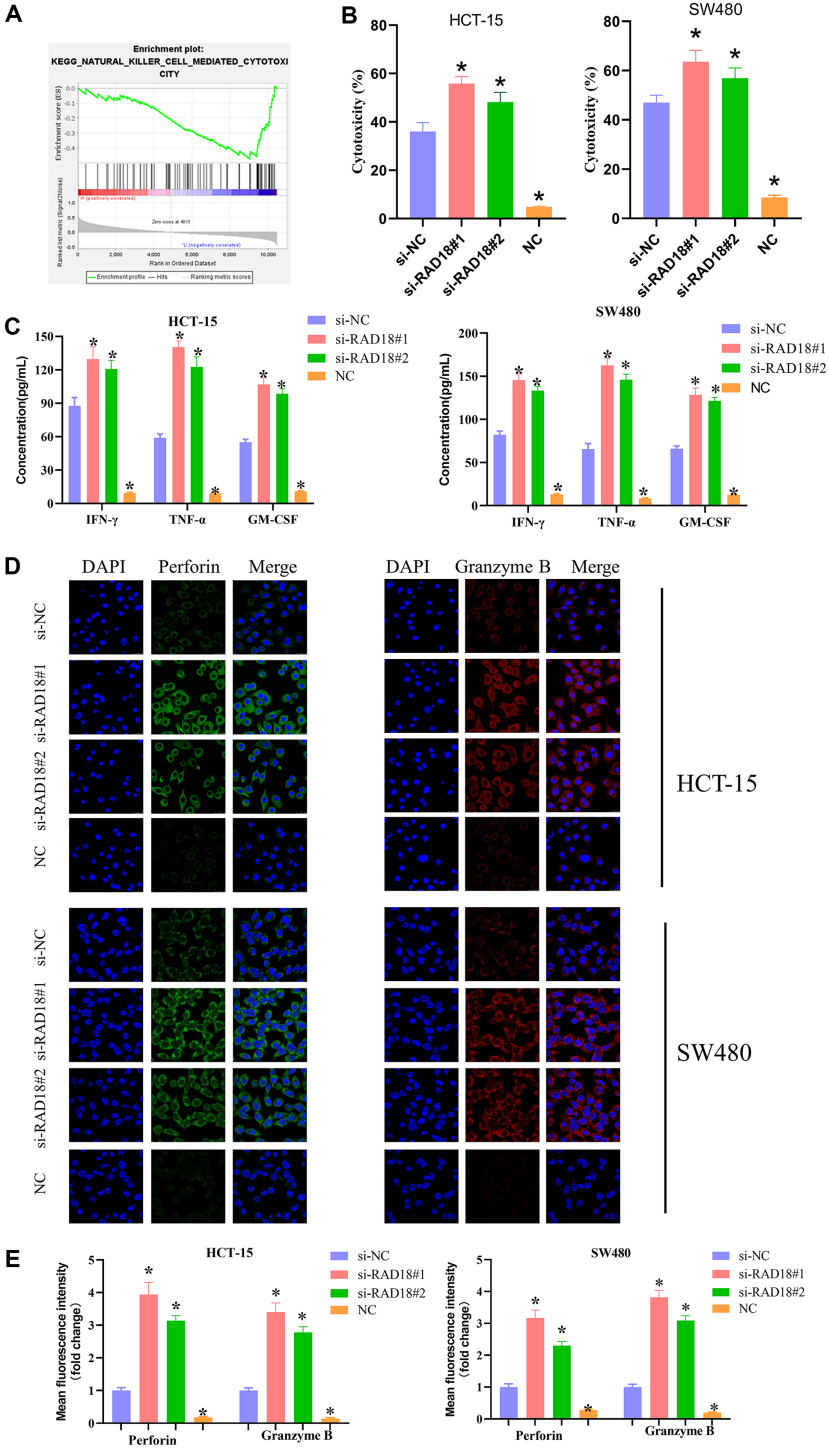
RAD18 hampers the killing effect of NK cells on CRC cells. (**A**) The enriched pathways of RAD18 were analyzed using GSEA database; (**B**) Cytotoxicity of NK cells was detected by LDH test; (**C**) Secretion levels of IFN-γ, TNF-α and GM-CSF in cells were detected by ELISA; (**D**) Expression of toxic molecules perforin and granzyme B was detected by IF; (**E**) Quantification of the mean fluorescence intensity of perforin and granzyme B. * means *p* < 0.05.

To explore enrichment pathways related to RAD18, enrichment analysis was performed by GSEA, and it was found that RAD18 was enriched in the NATURAL KILLER CELL MEDIATED CYTOTOXICITY pathway (Fig. 2A). We suggested that RAD18 may affect the malignant progression of CRC cells through the killing effect of NK cells. NK cell-mediated antitumor effects have been ascertained to depend on the presence of IL-2, which is a survival factor of NK cells and an enhancer of cytotoxicity [18]. We stimulated and activated NK92 cells with IL-2 (100 U/ml), constructed HCT-15 and SW480 cells with low RAD18 expression, and co-cultured with IL-2-activated NK92 cells to explore the effect of RAD18 on activated NK cells. In addition, we also examined the changes of HCT-15 and SW480 cells that were not co-cultured with activated NK92 cells and set them as negative control (NC) groups. As the LDH test revealed, NK cell cytotoxicity was greatly increased in the si-RAD18#1 group and si-RAD18#2 group. In contrast, CRC cells in the NC group only had very weak cytotoxicity themselves compared to the si-NC group (Fig. 2B). ELISA results of IFN-γ, TNF-α, and GM-CSF secretion showed that si-RAD18#1 and si-RAD18#2 significantly increased the secretion levels of IFN-γ, TNF-α, and GM-CSF of NK cells. The levels of IFN-γ, TNF-α, and GM-CSF in the NC group were significantly lower than those in the si-NC group (Fig. 2C). Additionally, the expression of toxic molecules perforin and granzyme B detected by IF assay revealed that the expression of perforin and granzyme B in si-RAD18#1 group and si-RAD18#2 group were greatly increased. In contrast to the si-NC group, the NC group had lower expression levels of perforin and granzyme B (Fig. 2D and 2E). These findings illustrated that RAD18 hampered NK cell-mediated cytotoxicity against CRC cells.

### E2F7 Is the Upstream Regulatory Molecule of RAD18

**Fig. 3:**
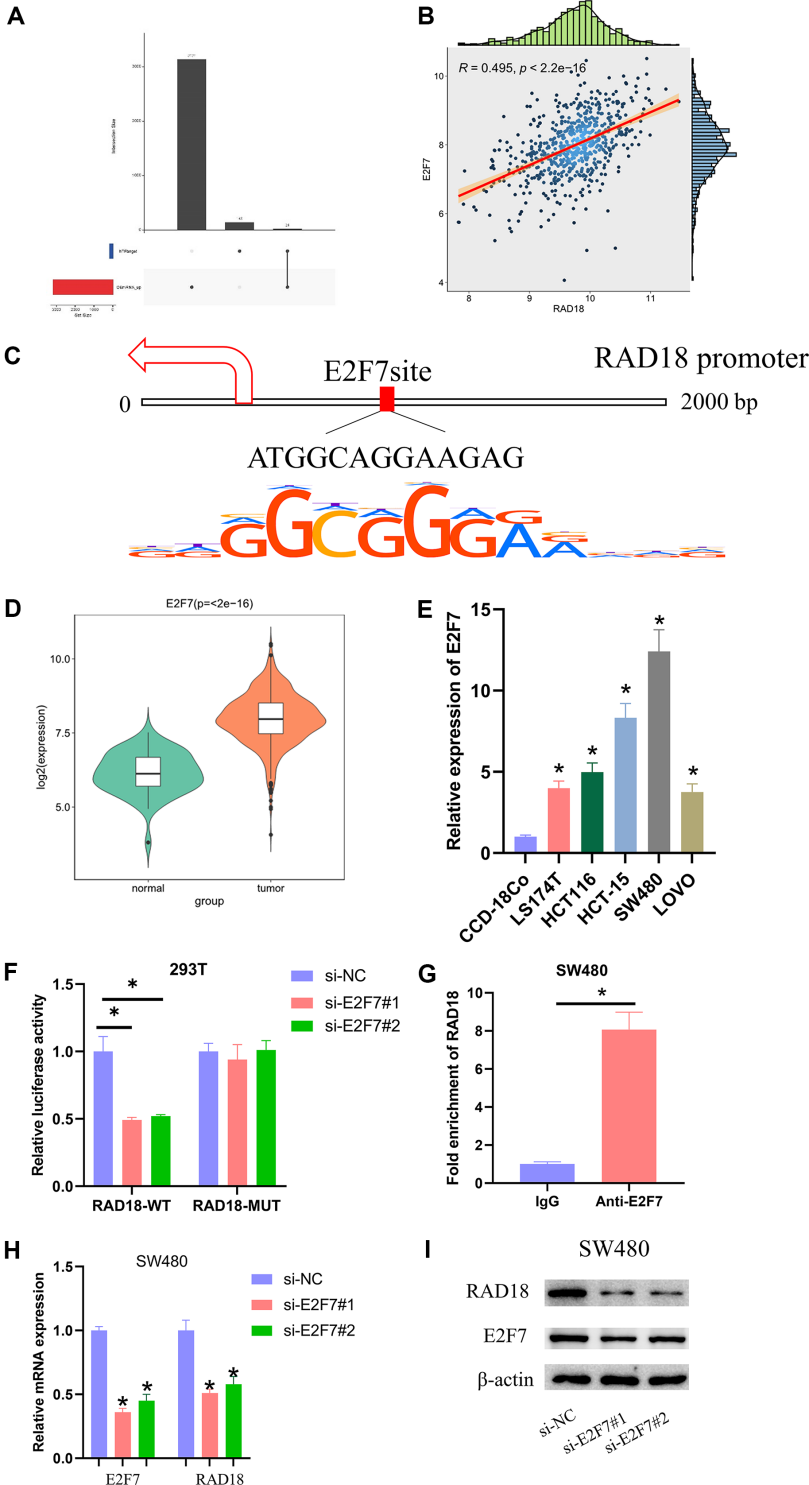
E2F7 activates the transcription of RAD18. (**A**) Upset plot of predicted upstream target genes and differentially expressed genes; (**B**) Pearson correlation analysis of E2F7 and RAD18; (**C**) Binding sites of E2F7 and RAD18 were analyzed by JASPAR database; (**D**) Expression of E2F7 in CRC tissues was analyzed by bioinformatics; (**E**) Expression of E2F7 in different cell lines was detected by qRT-PCR; (**F, G**) Dual-luciferase reporter assay and ChIP assay were used to verify the binding relationship between E2F7 and RAD18; (**H, I**) Expression of E2F7 and RAD18 was detected by qRT-PCR and western blot. * means *p* < 0.05.

To dive into the molecular mechanism of RAD18 regulating NK cell killing, we predicted upstream regulatory molecules of RAD18 by bioinformatics, and E2F7 was found positively correlated with the expression of RAD18 by correlation analysis (Fig. 3A and 3B). The query of transcription factor E2F7 using JASPAR revealed a potential binding site with RAD18 transcript at 2000 bp upstream (Fig. 3C). The significant up-regulation of E2F7 in CRC tissues and cells was found by bioinformatics and qRT-PCR assay (Fig. 3D and 3E). Among the above assays, the phenotype of SW480 was more obvious, so it was used in the following experiments. Then, dual-luciferase reporter assay showed that si-E2F7#1 and si-E2F7#2 could greatly reduce the luciferase activity of RAD18-WT without affecting RAD18-MUT (Fig. 3F). ChIP showed that Anti-E2F7 significantly increased RAD18 enrichment compared to IgG (Fig. 3G). The qRT-PCR and western blot results showed that si-E2F7#1 and si-E2F7#2 were able to significantly reduce the expression of E2F7 and RAD18 (Fig. 3H and 3I). The above experimental results revealed that E2F7 was an upstream regulatory molecule of RAD18.

### E2F7 Hampers the Killing Effect of NK Cells on CRC Cells by Activating RAD18 Transcription

**Fig. 4:**
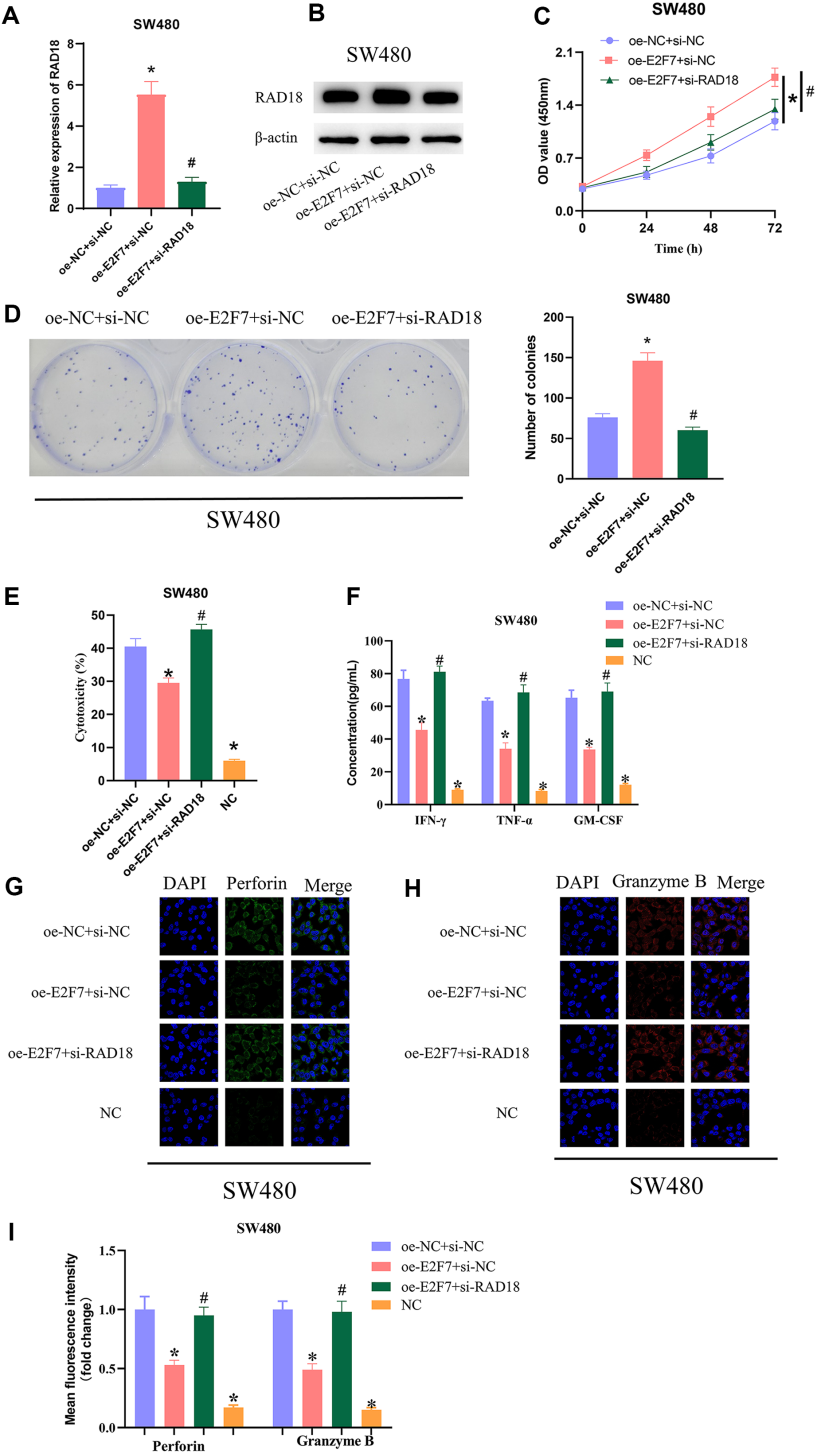
E2F7 hampers the killing effect of NK cells on CRC cells by activating RAD18 transcription. (**A**) qRTPCR was used to detect transfection efficiency; (**B**) Expression of RAD18 was detected by western blot; (**C**) Cell viability was detected by CCK-8 assay; (**D**) Proliferation ability of transfected cells was detected by colony formation assay; (**E**) Cytotoxicity of transfected NK cells was detected by LDH test; (**F**) Secretion levels of IFN-γ, TNF-α and GM-CSF in cells were detected by ELISA; (**G, H**) Expression of toxic molecules perforin and granzyme B were detected by IF; (**I**) Quantification of the mean fluorescence intensity of perforin and granzyme B. * vs oe-NC+si-NC, # vs oe-E2F7+si-NC, *p* < 0.05.

To investigate whether E2F7 inhibited NK cell killing by activating RAD18 transcription, we set up three different treatment groups (NC group: oe-NC+si-NC; E2F7 overexpression group: oe-E2F7+si-NC; co-transfection group: oe-E2F2+si-RAD18). As qRT-PCR and western blot results revealed, expression of RAD18 in the oe-E2F7+si-NC group was greatly up-regulated, and expression of RAD18 in the co-transfection group returned to the level of oe-NC+si-NC group (Fig. 4A and 4B). Cell viability was detected by CCK-8, with outcomes showing that cell viability was significantly up-regulated after E2F7 overexpression, and that in the co-transfection group returned to the level of oe-NC+si-NC group (Fig. 4C). Cell proliferation was assayed by colony formation assay, with results showing that oe-E2F7 could greatly promote cell proliferation compared with oe-NC+si-NC group, and co-transfection of si-RAD18 could reverse the promotion of E2F7 overexpression on cell proliferation (Fig. 4D). Subsequently, the cells after different transfection treatments were co-cultured with IL-2-activated NK92 cells, and a group of SW480 cells without co-culture with NK92 cells was set up as the NC group. The cytotoxicity of NK cells was assayed by LDH test, with results showing that the oe-E2F7+si-NC group had significantly reduced NK cell toxicity compared with the oe-NC+si-NC group, and further silencing of RAD18 could offset the inhibitory effect of the E2F7 overexpression on NK cell toxicity. While the NC group showed only weak cytotoxicity compared to the oe-NC+si-NC group (Fig. 4E). ELISA results showed that the secretion of IFN-γ, TNF-α, and GM-CSF by NK cells in the E2F7-overexpression group was largely lower than oe-NC+si-NC group, and further silencing of RAD18 could reverse the inhibitory effect of E2F7 overexpression on the secretion of IFN-γ, TNF-α, and GM-CSF by NK cells, whereas the levels of these cytokines were significantly low in SW480 cells from the NC group (Fig. 4F). Additionally, based on the expression of perforin and granzyme B detected by IF assay, the expression of perforin and granzyme B in oe-E2F7+si-NC group was greatly lower than those in the oe-NC+si-NC group, while in co-transfection group, it was restored to oe-NC+si-NC group level, which illustrated that silencing RAD18 reversed the effect of E2F7 overexpression on perforin and granzyme B expression. Also, the low expression of perforin and granzyme B in SW480 cells in the NC group suggested that both were mainly released by NK cells (Fig. 4G-4I). Together, these results proved that E2F7 hampered NK cell-mediated cytotoxicity against CRC cells by activating RAD18 transcription.

## Discussion

CRC covers about 10% of new cancer cases worldwide and is the second leading cause of cancer-related deaths [19, 20]. Recently, RAD18 has been ascertained to modulate proliferation, migration, and chemosensitivity of multiple cancers, including rectal cancer [21] and esophageal squamous cell carcinoma [22]. Overexpression of RAD18 is reported by Liu *et al*. [23] to induce DNA damage repair and foster 5-FU resistance in CRC cells. Li *et al*.[24] revealed up-regulation of RAD18 in CRC tissues that promoted cell invasion and migration. Here, our results showed high expression of RAD18 in CRC and promoted CRC cell viability and proliferation ability, consistent with previous studies.

Additionally, RAD18 was found to be enriched in the NATURAL KILLER CELL MEDIATED CYTOTOXI CITY signaling pathway by bioinformatics analysis. NK cells, named for their ability to autonomously kill target cells, are the master effector cells of cancer in innate immunity, highly heterogeneous in the microenvironment. In recent years, the killing effect of NK cells was ascertained to weigh heavily on eliminating tumor cells. For example, IL-2-activated NK cells can kill colon cancer cells effectively [25]. Abdelrahman *et al*. [26] reported that miR-182 induced NK cell cytotoxicity and upregulated perforin-1 secretion to enhance the cytotoxicity of NK cells against HCC cells. NK cells exert cytotoxic effects through a variety of mechanisms. Perforin and granzymes can be released by NK cells. Perforin creates pores on the surface of target cells, allowing granzyme B to enter and induce apoptosis. NK cells secrete a significant number of cytokines, such as IFN-γ and GM-CSF, which directly act on target cells or further activate other immune cells to attack the target cells. Furthermore, NK cells produce death ligands like TNF-α, which bind to death receptors expressed on the surface of target cells, thereby inducing cell death [27, 28]. Here, we found that silencing RAD18 enhanced NK cell cytotoxicity in CRC. At present, more and more research has focused on NK cell-based cancer immunotherapy. The correlation between NK cell toxicity reduction and metastasis has been confirmed in head and neck squamous cell carcinoma and pharyngeal cancer [29, 30]. In gastrointestinal sarcoma, NK cell infiltration is negatively correlated with metastasis [31]. These studies have shown that NK cells play an imperative role in anti-tumor immunotherapy. In summary, NK cell-based anti-tumor immunotherapy was expected to make a breakthrough in future clinical treatment.

It is worth noting that we also found that RAD18 had an upstream regulatory molecule E2F7. E2F7 is from the E2F family, which is widely expressed in various tissues and organs, and has been shown to regulate gene expression and participate in regulating cell proliferation, differentiation, DNA repair, and cell cycle [32]. E2F7 is reported to be a tumor promoter in BC, inducing cancer cell proliferation, invasion, and metastasis [33]. Yang *et al*. reported that E2F7 was up-regulated in glioblastoma patients, while its high expression was linked with poor overall survival. Functional studies have shown that E2F7 can promote cell proliferation, metastasis, and tumorigenicity [34]. Besides, studies have ascertained that E2F7 can be modulated by miRNA, affecting cancer progression. Guo *et al*. [35] discovered that miR-30a inhibited the malignant progression of papillary thyroid cancer cells by targeting E2F7 expression. In our study, we found that E2F7 was significantly up-regulated in CRC cells, fostered the malignant progression of cancer cells, and hampered the killing effect of NK cells on CRC cells by activating RAD18 transcription. The findings uncovered the function of E2F7 in promoting CRC development, hence E2F7 may be a novel target for CRC therapy.

The focus of this work was to confirm the essential role of RAD18 in CRC growth and identify a key upstream regulator of RAD18, E2F7, which reduced the killing effect of NK cells in CRC by activating RAD18 transcription. These findings proffered a broader perspective for understanding the pathogenesis of CRC. Nevertheless, limitations existed. Firstly, the inhibitory effect of RAD18 on NK cell killing CRC was not verified from the animal level. Secondly, the downstream regulatory mechanism of RAD18 was not involved. We intend to conduct in-depth excavation in the future to enrich the regulatory mechanism in the progression of CRC. Overall, our study not only enriched the regulatory mechanism of NK cells in tumors, but also laid a foundation for guiding NK cells for tumor immunotherapy, thus proffering a fresh target for clinical improvement of NK cell function. E2F7/RAD18 axis was ascertained by this study to be a feasible fresh therapeutic target for CRC.

## Figures and Tables

**Table 1:** qRT-PCR primer sequence.

Gene	Primer sequence (5’→3’)
E2F7	F: GGAAAGGCAACAGCAAACTCT
	R: TGGGAGAGCACCAAGAGTAGAAGA
RAD18	F: TAGCCTTCTCTATGTTGTCTATCCC
	R: TAGCCTTCTCTATGTTGTCTATCCC
GAPDH	F: CGACCACTTTGTCAAGCTCA
	R: AGGGGTCTACATGGCAACTG

**Table 2:** Primer sets for ChIP-qPCR assay.

Primer Sets	Primer sequence (5’→3’)
Site	F: TCTGTTCAGAGCAAGGACGG
	R: GCCGCTTAAGGGCTTGTACT
